# Dietary fibre enrichment of supplemental feed modulates the development of the intestinal tract in suckling piglets

**DOI:** 10.1186/s40104-019-0386-x

**Published:** 2019-10-08

**Authors:** H. M. J. Van Hees, M. Davids, D. Maes, S. Millet, S. Possemiers, L. A. den Hartog, T. A. T. G. van Kempen, G. P. J. Janssens

**Affiliations:** 1 Department of Nutrition, Genetics and Ethology, Ghent University, Merelbeke, Belgium; 2 Research and Development, Trouw Nutrition, Amersfoort, The Netherlands; 3 Department of Internal and Vascular Medicine, Amsterdam University Medical Centre, Amsterdam, The Netherlands; 4 Department of Reproduction, Obstetrics and Herd Health, Ghent University, Merelbeke, Belgium; 5 ILVO, Eenheid Dier, Melle, Belgium; 6 BioActor, Maastricht, The Netherlands; 7 Animal Nutrition, Wageningen University and Research, Wageningen, The Netherlands; 8 North Carolina State University, Raleigh, NC USA

**Keywords:** Dietary fibre, Gut maturation, Gut microbiota, Suckling piglets

## Abstract

**Background:**

Commercial pre-weaning diets are formulated to be highly digestible and nutrient-dense and contain low levels of dietary fibre. In contrast, pigs in a natural setting are manipulating fibre-rich plant material from a young age. Moreover, dietary fibre affects gastrointestinal tract (GIT) development and health in older pigs. We hypothesised that supplemental diets that contain vegetal fibres are accelerating GIT development in suckling piglets in terms of size and functionality. From d 2 of life, sow-suckled piglets had access to a low fibre diet (CON), a diet with a fermentable long-chain arabinoxylan (lc-AXOS), a diet with a largely non-fermentable purified cellulose (CELL), or a diet containing both fibres. During the initial 2 weeks, the control diet was a high-density milk replacer, followed by a dry and highly digestible creep meal. Upon weaning at 25 d, 15 piglets from each treatment group, identified as eaters and originating from six or seven litters, were sacrificed for post-mortem examination of GIT morphology, small intestinal permeability and metabolic profile of the digesta. The microbiota composition of the mid-colon was evaluated in a sub-set of ten piglets.

**Results:**

No major statistical interactions between the fibre sources were observed. Piglets consumed the fibre-containing milk supplements and creep diets well. Stomach size and small intestinal permeability was not affected. Large intestinal fill was increased with lc-AXOS only, while relative large intestinal weight was increased with both fibre sources (*P* < 0.050). Also, CELL decreased ileal pH and tended to increase ileal DM content compared to CON (*P* < 0.050). Moreover, the concentration of volatile fatty acids was increased in the caecum (*P* < 0.100) and mid-colon (*P* < 0.050) by addition of CELL. lc-AXOS only stimulated caecal propionate (*P* < 0.050). The microbiota composition showed a high individual variation and limited dietary impact. Nonetheless, CELL induced minor shifts in specific genera, with notable reductions of *Escherichia*-*Shigella*.

**Conclusions:**

Adding dietary fibres to the supplemental diet of suckling piglets altered large intestinal morphology but not small intestinal permeability. Moreover, dietary fibre showed effects on fermentation and modest changes of microbial populations in the hindgut, with more prominent effects from the low-fermentable cellulose.

## Introduction

A piglet is born with an immature digestive system that must go through major changes to digest and absorb solid food. Although partly following an innate developmental path, the gastrointestinal tract (GIT) displays a high plasticity and is able to respond to luminal factors, such as those originating from the diet and the establishing gut microbiota [1, 2]. Initially, colostrum and milk, and the bioactive components herein, will initiate development. Typically starting at the 2nd week of life, the GIT might be stimulated by solids when wild neonatal piglets follow the dam on her foraging trips, thus having access to all kinds of plant, animal and soil material. Indeed, observations in a semi-natural environment show that chewing straw and rooting is already performed during the second week of life [3, 4]. It is conceivable that this behaviour will result in intake of non-milk materials. In a commercial setting, suckling piglets are regularly offered supplemental diets in the form of milk replacers and dry feed (‘creep feed’) aiming to prepare the piglet for weaning. These diets are typically milk based, nutrient-dense and are formulated to be highly digestible and palatable. Commonly, they contain low levels of dietary fibre (DF) and may deviate largely from what neonatal wild pigs ingest. However, whether or not the early intake of DF is beneficial for GIT maturation in the pre-weaning piglet has received limited attention in scientific research.

As the pigs’ own enzymes cannot degrade DF, they require microbes harbouring in their intestines to utilize them. In contrast to the suckling piglet, the older pig’s microbiota has adapted to degrade the complex polysaccharides like arabinoxylans, pectin and, to a certain extent, cellulose present in plant cell wall. This allows the host to absorb and use the degradation products of fermentation, mainly volatile fatty acids (VFA), as energy substrate for maintenance and growth. Moreover, from post-weaning pig studies, it is known that DF is implicated in GIT development and health [5–7]. For instance, DF might stimulate the early development of the fibrolytic gut microbiota and the production of VFA [8]. The VFA, especially butyrate, stimulate gut epithelial cell proliferation and differentiation [9]. Furthermore, the inert, i.e. non-fermentable, fibre fraction might stimulate the gut wall through its bulking and abrasive effect and contribute to washing out pathogenic microbes [10]. Via these actions, DF might have an effect on the gut developmental trajectory. This may lead to a gut system, including its associated microbiota, which is readily prepared for post-weaning life where vegetal carbohydrates, fats and proteins will be the main nutrients.

Therefore, a study was conducted to investigate the effect of DF when added to milk- and dry creep feed diet supplements fed to suckling piglets. To this end, we selected a wheat arabinoxylan and a wood cellulose representing a fermentable and a non-fermentable fibre source, respectively. Both DF represent the main non-starch polysaccharides (NSP) found in cereal grains and other vegetable feedstuffs. Arabinoxylans belong to the hemicellulose fraction in grains and mainly consist of a backbone of the pentose xylose with arabinose side-chains. An array of non-host carbohydrate-active enzymes are needed for degradation of arabinoxylans, and therefore, they are assumed to be fermented post-ileum [11, 12]. Also, they are reported to have prebiotic properties in humanized rats [11] and piglets. For example, arabinoxylans can be utilized by fibrolytic *Bacteroides* spp. and *Prevotella* spp. which are dominant species in the adult pig colon [13]. Moreover, isolated arabinoxylans can decrease small intestinal and colonic permeability, lower caecal pH, increase VFA in the hindgut and modulate parameters of gut immunity [14, 15]. On the other hand, cellulose consists of tightly packed linear chains of glucose monomers (7–15 K monomers). This structural characteristic makes them poorly soluble and less readily used as a substrate for most gut bacteria, except those possessing cellulolytic enzymes [16]. High cellulose containing feedstuffs have been reported to alter stomach and large intestine (LI) development and to improve small intestinal barrier function, reduce the proliferation of pathogens and improve faecal consistency in post-weaning pigs [10, 17–19].

We hypothesised that supplemental diets enriched with both lc-AXOS and cellulose would stimulate the development of the GIT and its associated intestinal microbiota of the suckling piglet.

## Material and methods

The study was a 2 × 2 factorial design with four dietary treatments, i.e. diets with and without long-chain arabinoxylans (lc-AXOS) and cellulose (CELL).

### Animals and housing

Thirty-four Hypor Libra sows (Hendrix Genetics, Boxmeer, The Netherlands) of the resident herd of the research station were inseminated (Hypor Maxter) and moved to the farrowing unit 1 week prior to expected farrowing. The unit consisted of four climate-controlled rooms with ten farrowing crates (dimensions 200 cm × 260 cm) each. Rooms were lit from 06:00 h until 22:00 h. Sow dry feeders were elevated 40 cm above the floor hindering access by the piglets. No bedding material was provided. The sows were allowed to farrow spontaneously over a five-day period. Two sows with small litters were immediately weaned and their piglets redistributed over the remaining 32 litters. Litter size was equalised after 24 h while minimising cross-fostering. Piglets were ear-tagged to allow identification and processed following routine procedures, including an iron injection within 3 d after birth and vaccination against porcine reproductive and respiratory syndrome virus (Porcilis PRRS, MSD Animal Health) and F18 *E. coli* (Ecoporc Shiga, IDT Biologika GmbH). Neither teeth clipping nor castration were applied.

### Feeds and feeding

The basal composition of the milk supplement consisted of dairy ingredients, vegetal proteins and fats, synthetic amino acids and a vitamin and mineral premix. The test creep meals were based mainly on native and extruded cereals (corn, wheat, oats and barley), highly digestible vegetable proteins, whey powder, fish and vegetable oils, synthetic amino acids and a vitamin and mineral premix. The final diet was made with the basal mixtures to which extruded corn starch was added. The latter was exchanged on a *w*/*w* basis with the two model compounds (Tables 1 and 2). A purified finely ground wood cellulose powder was used as the first test compound (CELL; Arbocel BWW 40, Rettenmaier & Söhne, Rosenberg, Germany) and was characterised as having a low viscosity and a water-binding capacity of 6 g water per g DM. The second test compound was a low-viscous, water-extractable long-chain arabinoxylan oligosaccharide from wheat endosperm (lc-AXOS; BioActor, Maastricht, The Netherlands). Based on supplier information, it has a purity of at least 60% with a degree of polymerisation between 50 and 70.

**Table 1:** Composition of the experimental milk supplements^a^

	CON	lc-AXOS	CELL	lc-AXOS+CELL
Ingredient composition, %				
Basal milk supplement^b^	90	90	90	90
Chromium oxide (III)	0.3	0.3	0.3	0.3
Cellulose^c^			5.0	5.0
lc-AXOS^d^		2.0		2.0
Corn starch heat treated	9.7	7.7	4.7	2.7
Calculated nutrient composition, per kg				
ME, MJ	18.3	18.0	17.6	17.3
NE, MJ	14.7	14.4	14.1	13.9
Lys, g	16.0	15.9	15.9	15.8
Met+Cys, g	9.5	9.4	9.3	9.2
Thr, g	10.7	10.6	10.5	10.5
Trp, g	3.2	3.2	3.1	3.1
Starch (Ewers method), g	64	51	33	20
Lactose, g	348	348	348	348
Calcium, g	5.0	5.0	5.0	5.0
Phosphorus, g	4.8	4.7	4.6	4.6
Copper (total), mg	141	141	141	141
Zinc (total), mg	92	92	91	91
Analysed nutrient composition, per kg				
Moisture, g	40	38	36	34
Crude protein, g	212	213	213	212
Crude fat, g	195	194	198	193
Ash, g	65	64	65	65
NDF, g	34	26	57	60

^a^Piglets were fed supplemental milk diets (water:powder ratio 2.5:1) from d 2 to 13. From d 14 to 16 milk was gradually replaced by dry creep meals which were fed until weaning

^b^Basal milk supplement consisted of dairy whey products (63.3%), fats and oils (20.0%), wheat protein (5.6%), dextrose (5.0%), soy protein (2.8%), synthetic amino acids (1.3%), vitamin and mineral premix (1.0%) and organic acids (1.0%)

^c^Arbocel® BWW, natural pure cellulose (J. Rettenmaier & Sohne GmbH, Rosenberg, Germany)

^d^Naxus, long-chain arabinoxylans extracted from wheat endosperm (BioActor B.V., Maastricht, The Netherlands)

**Table 2:** Composition of the experimental dry creep meals^a^

	CON	lc-AXOS	CELL	lc-AXOS+CELL
Ingredient composition, %				
Basal meal ^b^	91.0	91.0	91.0	91.0
Chromium oxide (III)	0.3	0.3	0.3	0.3
Cellulose^c^			5.0	5.0
lc-AXOS^d^		2.0		2.0
Corn starch heat treated	8.7	6.7	3.7	1.7
Calculated nutrient composition, per kg				
ME, MJ	7.5	7.2	6.8	6.5
NE, MJ	11.5	11.2	10.9	10.7
Lys, g	15.7	15.6	15.6	15.5
Met+Cys, g	9.0	8.9	8.8	8.8
Thr, g	10.2	10.1	10.0	10.0
Trp, g	3.1	3.1	3.1	3.1
Starch (Ewers method), g	241	229	210	198
Lactose, g	145	145	145	145
Calcium, g	5.2	5.2	5.2	5.2
Phosphorus, g	5.9	5.8	5.7	5.7
Copper (total), mg	176	176	176	176
Zinc (total), mg	101	100	100	99
Analysed nutrient composition, per kg				
Moisture, g	65	65	64	62
Crude protein, g	188	189	186	185
Crude fat, g	125	123	119	118
Ash, g	50	51	50	50
NDF, g	60	67	114	107

^a^Piglets were fed supplemental milk diets (water:powder ratio 2.5:1) from d 2 to 13. From d 14 to 16, milk was gradually replaced by dry creep meals which were fed until weaning

^b^Basal meal consisted of heat-treated cereals (29.1%), mono- and disaccharides (17.2%), dairy whey products (16.5%), extruded soybean meal (12.6%), heat-treated soy beans (6.78%), vegetable proteins (5.56%), barley (4.44%), fats and oils (3.56%), vitamins and minerals (1.94%), synthetic amino acids (1.79%) and organic acids (0.53%)

^c^Arbocel® BWW natural pure cellulose (J. Rettenmaier & Sohne GmbH, Rosenberg, Germany)

^d^Naxus, long-chain arabinoxylans extracted from wheat endosperm (BioActor B.V., Maastricht, The Netherlands)

Dose-response relations for DF and its effect on zootechnical or GIT parameters are not established for suckling pigs. The lc-AXOS was included at 2% because of the relatively low feed intake of suckling piglets. The CELL was included at 5%, which was extrapolated from the levels used by Metzler-Zebeli et al. [20] and Chen et al. [14]. The combination supplemental diet contained 2% lc-AXOS and 5% CELL. This resulted in four final milk supplements and four creep meals that were only marginally different in energy and protein content. Organic acids were added to both the milk supplement and the creep meal as preservatives while palatants were added to increase attractiveness. Chromium oxide (0.3% *w*/*w*) was added as a faecal colour marker. No other feed additives were used. Moreover, all test diets were formulated to meet or exceed the nutrient requirements for this category of pigs.

Upon entering the farrowing unit, sows were fed a lactation diet (ForFarmers, The Netherlands; 9.0 MJ NE per kg, 156 g crude protein and 8.2 g lysine per kg). Once born, their piglets were allowed to suckle freely. Supplemental milk was freshly prepared at least three times per day from milk powder mixed with water (1 kg milk powder:2.5 kg water) and provided from experimental d 2 until d 13. Then, from d 14 to 16, the milk was gradually replaced by dry creep meal, which was fed until weaning. Supplemental feeds were available ad libitum in round feeders (diameter 27 cm) designed for suckling piglets. Water was always available through nipple waterers specifically suited for sows or for suckling piglets.

### Sampling and measurements

Individual body weight of piglets was measured at birth, 24 h, d 13 and at weaning on d 25. Piglet weight increments during the first 24 h were used to estimate the colostrum intake. Daily supplemental diet disappearance was recorded per litter. In order to identify piglets that actually were consuming supplemental diets, faecal swabs were taken on d 6, 13,19 and 22 and checked for the presence of the green dye as previously described [21]. ‘Eaters’ were those animals observed to have green coloured faeces on d 13 and 19 or d 22. Prior to weaning (i.e. d 23 and 24), 15 clinically healthy and normal growing piglets per treatment were selected for sampling. These piglets were also required to meet the following additional criteria: designated as ‘eater’, average (within ±1 SD of the mean) birth weight and colostrum intake. Piglets were euthanized by an intra-cardiac injection containing 40% barbiturate pentobarbital, and a midline laparotomy was performed immediately thereafter to gain access to the gastrointestinal tract. The stomach was removed and weighed, both full and emptied. The small intestine (SI) was cut at the ileo-caecal junction and prepared free from its mesentery ligaments and its length and weight was recorded. Subsequently, 20 cm of the most distal part and a 20-cm section at 25% of the proximal SI was collected on ice-mounted petri discs for the everted sac procedure (described below). The remainder of the SI was emptied by gently squeezing contents into a container. From this, a representative sample was taken and stored for pH, VFA and ammonia analysis. The LI was cut at the rectum, prepared free from mesentery ligaments, weighed (empty and full) and its length was taken. Digesta samples from the caecum and at 50% of the LI length were snap frozen for microbiota profiling, pH measurement, VFA and ammonia analysis.

### Gut content metabolic profile

Homogenous 1 g digesta samples were diluted with 2 M sulfuric acid, thoroughly mixed and centrifuged. The supernatant was analysed for lactic acid and VFA by HPLC on a BioRad Aminex HPX-87H using a 0.005 mol/L sulfuric acid eluent at a flow rate of 0.7 mL/min. Ammonia-N (nitrogen as NH_4_^+^ and NH_3_) was analysed colourimetrically using the Berthelot reaction. Briefly, the sample was deproteinated using trichloroacetic acid and then chlorinated with sodium hypochlorite under alkalic conditions. This resulted in the conversion of NH_3_ to chloramine (NH_2_Cl). Subsequently, indolphenol and sodium nitroprusside were added to form indolphenol blue [22]. The absorbance was measured in microtitration plates at 630 nm (SpectraMax M2, Molecular Devices, San Jose, CA, USA) and compared to a standard curve.

### Gut permeability

The everted sac procedure was used to assess gut permeability of the proximal and distal SI as described by De Greeff et al. [23]. Briefly, a 20 cm gut section was cleaned with PBS, everted and filled with a 5 mmol/L glucose-PBS solution and submerged in PBS kept aeriated and at 39 °C. The submersion fluid also contained permeability markers, i.e. patent blue (Mol. weight 583; 3.6 g/L) and Co-EDTA (Mol. weight 347; 40 g/L). Both markers are assumed to permeate para-cellularly based on their molecular weight. They were analysed in a 10-mL sample extracted from the gut segment after 1 h of incubation. The Patent Blue concentration was determined spectro-photometrically (SpectraMax M2, Molecular Devices, San Jose, CA, USA) at a wave-length of 640 nm. Cobalt was analysed by inductively coupled plasma-mass spectrometry (NexION 350D, PerkinElmer Inc., Waltham, MA, USA).

### Microbiota analysis based on 16S rRNA

From the 15 animals sacrificed per treatment, a subset of ten randomly chosen individuals was used for microbiota analysis. To this end, a representative luminal sample from the mid-colon was taken, immediately snap frozen on dry ice and then transferred to a − 80 °C freezer. Subsequently, cell lysis was performed (MagNA Lyser; Roche, Burges Hill, UK) and genetic material was extracted using the MO BIO (Carlsbad, CA, USA) PowerMicrobiome™ RNA isolation kit following the manufacturer’s instructions with a few modifications, i.e. by omitting the β-mercaptoethanol and DNase I. Mid-colon 16S rRNA gene libraries were prepared by amplification of the V3–4 regions as described by Kozich, Westcott [24] with some modifications. For example, to reduce PCR bias in high template samples,12.5 ng bacterial DNA was used as template in the PCR with KAPA HiFi Hotstart ReadyMix (Kapa Biosystems, Woburn, MA, USA). Equimolar amounts of the correctly sized fragments were pooled for sequencing. The pool was run on an agarose gel and the amplicon was extracted from the gel and purified by QIAquick Gel Extraction Kit (Qiagen, Hilden, Germany). The library was sequenced on an Illumina HiSeq platform 2 × 300 paired end. Sequence data was processed and annotated using mothur (version 1.39 [25]). 100 K reads per sample were merged and quality-filtered against ambiguous bases and short fragments (< 400 bases). Next, sequences were de-replicated and aligned against the SILVA database NR-123 [26]. Only sequences that aligned from positions 6428 to 23,440 were retained while others were trimmed. Sequences were pre-clustered, and chimeras were removed. Vsearch was used to cluster the final sequence set into operational taxonomic units (OTUs) at 97% similarity. Taxonomy was assigned using the Ribosomal Database Project Classifier (RDP; [27]), and sequences classified as unknown, chloroplast, mitochondria, Archaea or Eukaryotes were removed.

### Statistical analysis

No major interactions between CELL and lc-AXOS were observed (*P* < 0.010). Hence, the main effects are reported herein. The zootechnical parameters were evaluated using the PROC GLM procedure of SAS Studio (SAS Institute Inc., Cary, NC, USA) with treatment and farrowing room as class variables. Diet and day of euthanasia were class variables to evaluate the effect on gut metrics while individual piglets were nested within a sow-litter combination using the following model:

$$ {\mathrm{Y}}_{\mathrm{i}\mathrm{jkl}}=\upmu +{\mathrm{day}}_{\mathrm{i}}+\mathrm{lc}-{\mathrm{AXOS}}_{\mathrm{j}}+{\mathrm{CELL}}_{\mathrm{k}}+\mathrm{Sow}\left(\mathrm{CELL},\mathrm{lc}-\mathrm{AXOS}\right)+{\mathrm{e}}_{\mathrm{i}\mathrm{jkl}} $$

where Y_ijk_ = dependent variable, μ = overall mean, day = sampling day (i = 23,24), dietary treatments, i.e. lc-AXOS (0 or 2%) and CELL (0 or 5%) and e_ij_ = residual error. Differences were considered significant if *P* < 0.050 and 0.05 < *P* ≤ 0.100 was considered a trend.

Microbiota data analysis were performed in R using vegan (vegan: Community Ecology Package, R-package version 2.4–3) and phyloseq [28]. We used permutation ANOVA to determine the correlation between the microbial composition and other gut and animal performance parameters. Deseq2 was used to test differential abundance of the OTUs between treatments [29].

## Results

Generally, clinical health of the animals during the study was good. The supplemental feed intake showed a high inter-litter variation and a typical pattern of low intakes during the initial days after birth followed by a gradual increase up to 2 weeks and a steep rise during the week prior to weaning. Prior to weaning, 77% of the piglets were consuming the supplemental diets, and this was the same for all treatment groups (Table 2). Supplemental milk intakes were higher for CELL and the combination of lc-AXOS and CELL when compared to CON (*P* = 0.018), while DM intakes from creep meal were not different (*P* = 0.108; Table 3). Additional zootechnical data are presented in Table 3.

**Table 3:** Zootechnical data of animals under study. Data expressed as means

Parameter	CON	lc-AXOS	CELL	lc-AXOS×CELL	Pooled SEM	*P*-value
Number of sows	7	7	6	6	–	–
Parity (range)	3.0 (1–6)	3.4 (1–6)	4.0 (2–6)	3.3 (1–5)	0.69	0.857
Litter size after cross fostering	12.9	13.1	13.2	13.3	0.26	0.638
Litter size at weaning	12.3	13.1	13.0	12.7	0.31	0.215
Boar/gilt ratio	8/7	7/8	7/8	6/9	–	–
Birth	1.42	1.62	1.64	1.52	0.118	0.713
D 13	4.97	4.81	4.91	4.80	0.300	0.776
End (d 23/24)	7.99	7.87	8.25	7.80	0.438	0.817
Estimated colostrum intake, g	443	519	515	463	44.7	0.662
Milk supplement intake, g DM^1^	218^a^	253^ab^	342^b^	346^b^	43.4	0.018
Creep meal intake, g DM^1^	369	468	649	555	95.5	0.108
Ratio eaters:non-eaters^2^	0.76	0.77	0.73	0.80	0.082	0.930

^1^Estimate based on DM intake per litter divided by litter size at weaning. ^2^In litters used for the study. ‘Eaters’ were animals that had green coloured faeces on d 13 and 19 or d 22. ^a,b^ Values with different superscripts within a row are significantly different (*P* < 0.05)

### Gastrointestinal morphometrics

Fibre source did not affect stomach weight (Table 4). The SI tended to be longer with CELL (*P* = 0.080). The SI permeability was not affected by fibre source (*P* > 0.100). The LI contained more digesta with fibrous diets (*P* = 0.006 and 0.082, for lc-AXOS and CELL, respectively). Moreover, CELL increased LI length (*P* = 0.019), and both fibres increased relative LI weight expressed as percentage of body weight (*P* = 0.026 and 0.021 for lc-AXOS and CELL, respectively). SI and LI weight:length ratio did not differ between treatments.

**Table 4:** Gastrointestinal morphometrics and *ex-vivo* small intestinal permeability. Data are expressed as LSmeans (*n* = 15 per treatment)

Parameter	Lc-AXOS		CELL		Pooled	*P*-value	
	No	Yes	No	Yes	SEM	lc-AXOS	CELL
Absolute metrics							
Stomach weight, g	45	44	43	45	2.0	0.606	0.572
Small intestine length, cm^a^	746	771	728	789	23.7	0.408	0.080
Small intestine weight, g ^a^	219	222	219	222	9.5	0.746	0.789
Large intestine length, cm	132	136	127	140	3.6	0.347	0.019
Large intestine weight, g	64	71	64	71	2.8	0.123	0.108
Large intestine fill, g	56	76	60	72	4.6	0.006	0.082
Weight relative to body weight, %							
Stomach	0.56	0.57	0.54	0.58	0.020	0.726	0.178
Small intestine	2.71	2.87	2.72	2.86	0.096	0.223	0.334
Large intestine	0.80	0.92	0.80	0.92	0.035	0.026	0.021
Weight: length ratio, g/cm							
Small intestine (SI)	0.30	0.29	0.30	0.28	0.011	0.657	0.201
Large intestine	0.50	0.52	0.51	0.51	0.021	0.480	0.910
SI permeability, log_10_ mg/L ^b^							
Proximal (PB)	1.18	1.05	1.14	1.09	0.074	0.246	0.699
Proximal (Co)	1.97	1.87	1.94	1.90	0.054	0.219	0.631
Distal (PB)	0.99	0.99	0.97	1.02	0.069	0.855	0.587
Distal (Co)	1.84	1.83	1.82	1.85	0.045	0.896	0.557

^a^Excluding the gut sections used for the everted sac procedure

^b^Data are log_10_ transformed concentrations of Patent Blue (PB) and cobalt (Co) in the everted sac section

### Microbiota and metabolic profiles

The DM concentration tended to increase (*P* = 0.064) and pH of the ileum content for piglets fed CELL decreased (*P* = 0.030). Dietary fibres did not alter DM and pH in the more distal gut (Table 5). In the caecum, CELL tended to increase acetic acid (*P* = 0.073) and total VFA (*P* = 0.092) concentration, while lc-AXOS increased propionic acid (*P* = 0.030). In the mid-colon, concentrations of acetic- and butyric acid and total VFA were increased by CELL (*P <* 0.050), while lc-AXOS elicited no significant changes.

**Table 5:** Bacterial metabolites (mmol/L), pH and dry matter in different sections of the gut. Data are expressed as LSmeans (*n* = 15 per treatment)

Parameter	lc-AXOS		CELL		Pooled	*P-*value	
	No	Yes	No	Yes	SEM	lc-AXOS	CELL
Ileum							
Dry matter, kg/kg	0.17	0.14	0.15	0.18	0.011	0.501	0.064
pH	6.16	6.28	6.37	6.07	0.091	0.355	0.030
Ammonia-N	8	7	9	7	0.9	0.277	0.135
Lactate	6	7	6	7	0.9	0.178	0.202
Acetic acid	4	3	4	3	0.6	0.534	0.245
Propionic acid	bd^1^	bd	bd	bd	–		
Butyric acid	bd	bd	bd	bd	–		
Valeric acid	bd	bd	bd	bd	–		
Branched-chain VFA^2,3^	5	4	4	5	0.5	0.166	0.479
Total VFA^4^	10	8	10	9	0.8	0.248	0.725
Caecum							
Dry matter, kg/kg	0.13	0.12	0.12	0.13	0.005	O.837	0.921
pH	6.10	6.09	6.11	6.08	0.041	0.784	0.547
Ammonia-N	58	55	58	55	3.2	0.386	0.491
Lactate	bd	bd	bd	bd	–		
Acetic acid	72	74	69	77	3.2	0.770	0.073
Propionic acid	22	25	23	24	1.1	0.030	0.451
Butyric acid	12	12	12	12	0.7	0.914	0.559
Valeric acid	2	2	2	2	0.3	0.813	0.952
Branched-chain VFA^3^	5	5	4	5	0.4	0.886	0.651
Total VFA^4^	114	118	111	121	3.9	0.475	0.092
Mid-colon							
Dry matter, kg/kg	0.17	0.18	0.18	0.17	0.011	0.523	0.653
pH	6.17	6.15	6.20	6.12	0.051	0.769	0.314
Ammonia-N	62	62	62	62	2.2	0.861	0.975
Lactate	7	5	4	8	1.6	0.599	0.057
Acetic acid	44	44	35	53	4.4	0.955	0.007
Propionic acid	10	13	10	13	1.4	0.187	0.238
Butyric acid	15	15	13	16	0.8	0.871	0.021
Valeric acid	1	1	1	1	0.2	0.180	0.982
Branched-chain VFA^3^	4	5	5	5	0.5	0.401	0.881
Total VFA^4^	74	77	64	87	6.5	0.642	0.024

^1^ *bd* = below detection limit; ^2^ Iso-butyric and iso-valeric acid; ^3^ Mainly iso-butyric as iso-valeric acid was below detection limit; ^4^ *Total VFA* = sum of acetic, propionic, butyric, valeric and branched chain VFA

**Fig. 1:**
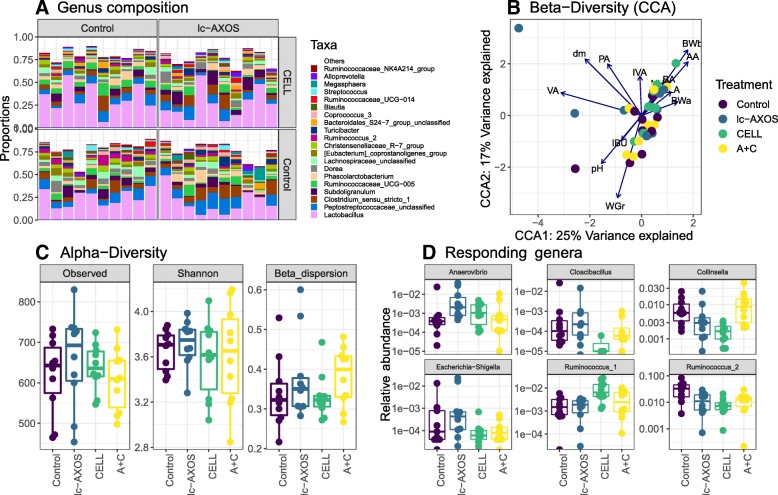
**a**-**d** Microbiota composition analysis of the mid-colon. CELL = diet enriched with purified cellulose, lc-AXOS = diet enriched with long-chain arabinoxylans (*n* = 10 per treatment). **a** Proportions of the 20 most abundant genera in the mid-colon per dietary treatment and animal. **b** Constrained Correspondence Analysis (CCA) of the Bray-Curtis beta-diversity ordinated against zootechnical parameters and bacterial metabolites in the mid-colon. AA; Acetic acid, BA; Butyric acid; PA; Propionic acid, LA; Lactic acid; VA; Valeric acid, IBA; Isobutyric acid, IVA; Isovaleric acid, dry matter, BWa; Body weight at autopsy; BWb Body weight at birth; WGr; weight gain relative to BWb. **c** Diversity metrics, i.e. observed diversity in OTUs, Shannon index and Beta-dispersion index on Bray-Curtis None of them were significantly different (*P* > 0.100). **d** Relative abundance of six genera with a significant (*P* < 0.05) differential abundance in response to dietary treatments

In general, the most abundant OTUs belonged to the Firmicutes and Bacteroides phyla covering over 95% of the sequences, followed by Actinobacteria and Spirochaetes. Within Firmicutes, the classes Clostridia and Bacilli were highly represented in all samples. The core microbiota on genus level revealed a large individual variation within all groups (Fig. 1a). While treatments did not show a shift in alpha diversity (expressed as Shannon-index) or Bray-Curtis beta-diversity (Fig. 1b and c), variance of Shannon diversity was increased for the piglets fed CELL (*P* = 0.007). An increased variance was not observed for between-sample diversity (beta-dispersion; Fig. 1b). The constrained correspondence plot (CCA) did not reveal associations between treatments and microbiota composition, GIT or performance parameters, other than associations between pH and relative daily gain (g body weight gain per kg of birth weight) versus the organic acid concentrations (Fig. 1b). Differential abundance tests showed various taxa responding to CELL, most markedly by the changes in two genera belonging to the family of Ruminococcaceae and a reduction in the *Escherichia-Shigella* genus (Fig. 1d).

## Discussion

Our data suggest that dietary fibres (DF) stimulate aspects of large intestinal development in suckling piglets. Unlike most other studies, piglets utilized for dissection were verified to consume the test diets. This is an important experimental aspect as up to one quarter of piglets were reported to not eat solid diets prior to weaning [21, 30], as was the case in the current study. The fibre-containing supplemental diets were well consumed when compared to the control diet as indicated by greater supplemental feed intake and large intestinal fill. This is consistent with observations by Hanczakowska et al. [31] and Zhang et al. [8]. Yet, despite the fact that the observed intakes on litter level are similar or higher compared to literature reports (e.g. [8, 21, 30]), the paucity of significant differences may have been caused by the low supplemental feed intake. Moreover, the experimental design did not allow the quantification of individual nutrient intakes, which are known to be variable [32].

Clearly, another complexing factor when studying the effect of supplemental diets in the suckling piglet is the fact that sow milk consumption will have a significant yet unquantifiable impact on gut development, its bacterial community and its metabolic activity. For instance, sow milk contains a suite of bioactive compounds, such as growth factors (e.g. epidermal growth factor, insulin like growth factor) and immunoglobulins. In addition, it contains a plethora of different oligosaccharide structures (porcine milk oligosaccharides; PMO, [57, 58]) that are substrates for fermentation by gut microbes. Indeed, sow milk composition varies between individuals and can shape the microbiota composition [34]. Compared with data in sow milk fed piglets [59, 60], in the current study VFA levels of the control animals seemed to be stimulated in the caecum and colon section of the gut. This is indicative of a background stimulation brought about by the basal diet. The above suggests that background substrate levels may already be present at considerable concentrations, and that their profile – and how to modulate that – deserves more attention in the light of gut maturation strategies.

### Effect of dietary fibre on large intestinal microbiota and metabolites

In general, the effect of DF on gastrointestinal function and development are primarily ascribed to alterations in the gastrointestinal microbial composition and its metabolic activity*,* i.e. VFA production. The GIT microbiota of the neonatal piglet under commercial conditions is shaped by the sow vagina, manure, milk and the pen flooring in interaction with the host genome [33]. The introduction of supplemental feeds was shown to be another source of influence [34]. However, enrichment with DF of the supplemental feeds did not lead to major changes in the microbiota composition. In our experiment, animals that were consuming the supplemental diets from experimental d 13 onwards were used, while we observed only a few of them to eat on d 6. This implies that consumption of diets occurred for at least 10 d prior to the time of the measurements. Still, this period may have been too short for the adaptation of the intestinal microbiota to fully ferment NSP, especially a more structurally complex type. It has been suggested that a period of 14–21 d in pigs over 25 kg [35] or even longer in the young pig [36, 37], is needed for full adaptation. Moreover, individual differences in adaptation time to DF in microbial composition have been reported [38]. Indeed, the observed increased variance in diversity of the mid-colon microbial composition may reflect an individual response, whether or not caused by the underlying variation in feed and milk intake pattern. However, this hypothesis is not supported by the variance in beta diversity.

Only a few literature reports exist on pre-weaning DF induced microbial shifts. For instance, Zhang and co-workers observed shifts in specific groups with fibre-enriched diets when compared to a low-fibre control diet [8]. In a follow-up paper, using qPCR, they were able to demonstrate an upward trend in *Clostridium* cluster XIVa and genes for the butyrate pathway with cellulose [39]. This is consistent with an increase in colonic butyrate concentration by cellulose observed in the present study. Moreover, shifts in the metabolic profile in the lumen of the hindgut were detected with CELL. This suggested the stimulation of cellulolytic microbes, as was earlier shown in the older pig [40, 41]. The observed shifts within Ruminococcaceae do support this notion (Fig. 1d). Alternatively, cellulose may have increased the transit of substrate through the digestive tract, thus reducing the time for pre-caecal digestion and absorption [42, 43]. Moreover, DF and particularly insoluble fibre sources lead to increased ileal endogenous nitrogen losses [44]. As a result, more nutrients*,* e.g. starch, proteins but perhaps also lactose, are arriving in the hindgut to become substrate for the microbiota. Indeed, the higher DM and lower pH does point toward more substrate reaching the end of the SI. Also, the increased concentrations of acetate, butyrate and lactate in the hindgut, indeed indicate a general stimulation of microbial fermentation and this is consistent with increased colonic fill and size. The increased LI size observed with the arabinoxylans might be attributed to the increase in digesta volume and less through the stimulatory effect of VFA.

Based on several studies, stimulation of specific groups of gut bacteria e.g. *Lactobacillus* spp. [14] and *Bifidobacterium*, *Bacteriodes* and *Roseburia* [45, 46] by lc-AXOS were expected. Partly, this lack of incongruence with earlier work may be explained by differences in the research model used, the composition of the basal diet and the DF inclusion level. Still, a slight but significant increase in caecal propionic acid concentration was observed, which is in agreement with earlier reports [11, 14, 46].

### Dietary fibre enrichment and indicators for intestinal health

Gut permeability is regarded as an important maturational aspect because a more permeable gut could lead to translocation of toxins and pathogens, causing a health risk for the animal. In young piglets receiving only sow milk, a reduction of marker passage over the SI wall with age was observed [47], and the same group showed that diet might modulate this parameter [48]. In the post-weaning pig fed a synthetic low-fibre basal diet enriched with lc-AXOS, Chen and co-workers reported reduced SI and colonic permeability. Still, this was only true for a high molecular weight marker and not for a smaller-sized marker molecule, indicating an effect on transcellular permeability only [14]. The same authors ascribed the main gut barrier enhancing properties of wheat bran to the arabinoxylans and not to the cellulose fraction. In contrast, we were not able to confirm a reduction of small intestinal permeability with either of the two model fibres. Similarly, the study by De Greeff et al. [23] showed that supplemental feeding stimulated several parameters of gut development but not gut barrier function.

During the initial post-natal weeks, the microbiota is variable and prone to perturbations. In this phase, intestinal pathogens start to proliferate and can cause clinical disease [49]. The observed stimulation of VFA and lactate in the hindgut with cellulose may be regarded as beneficial as it can inhibit the growth of bacterial pathogens like *E. coli* [50, 51]. Specifically, butyrate is regarded as important for the maintenance of the gut barrier function, since it is the main energy source for colonocytes [9]. Furthermore, the abrasive effect of the insoluble cellulose may cause sloughing of epithelial mucus together with the adherent microbes. This lowers the opportunities for potential pathogens from the Enterobacteriaceae family to proliferate [10, 52, 53] and agrees with the reduction of the closely related and potentially pathogenic genera *Escherichia-Shigella* in our data.

Branched-chain fatty acids (BCFA) and ammonium concentrations reflect bacterial protein breakdown and are irritants for gut epithelium [54]. Despite earlier reports that DF can reduce putrefactive fermentation [50], we were not able to confirm this in our study. This suggests an unaltered protein:carbohydrate ratio of the substrate available for fermentation by the colonic microbiota.

The increased large intestinal fill caused by DF may lead to shorter digesta transit time reducing the risk of constipation [55]. It is currently not clear, however, what the clinical relevance is for suckling pigs. A more developed LI can lead to an increased resorption of water and electrolytes, thus lowering faecal fluid losses and the risk of faeces inconsistency after weaning, as suggested by van Beers-Schreurs [56]. However, at the mid-colon level the DM concentration was not altered in our study.

Finally, it remains to be elucidated whether the magnitude of the observed changes due to DF are able to elicit health and performance benefits when piglets are subjected to weaning.

## Conclusions

The suckling piglets accepted the fibre-enriched supplemental diets. In support of our hypothesis, addition of dietary fibres increased large intestinal size and fill. VFA production in the hindgut was stimulated mainly by cellulose. Effects on mid-colon microbial composition were absent, except for some minor shifts in specific genera with cellulose.

## Data Availability

Detailed microbiota analytical workflow and additional data can be found at: https://github.com/AMCMC/Trouw_S16404. Other datasets generated and/or analysed during the current study are available from the corresponding author upon reasonable request.

## Abbreviations

BCFA: Branched-chain fatty acids
CELL: Cellulose
DF: Dietary fibre
GIT: Gastrointestinal tract
lc-AXOS: Long-chain arabinoxylans
LI: Large intestine
NSP: Non-starch polysaccharides
OTU: Operational taxonomic units
RDP: Ribosomal database project classifier
SI: Small intestine
VFA: Volatile fatty acids
